# Do Endogenously Produced and Dietary ω-3 Fatty Acids Act Differently?

**DOI:** 10.1093/function/zqad009

**Published:** 2023-02-23

**Authors:** Philip C Calder

**Affiliations:** School of Human Development and Health, Faculty of Medicine, University of Southampton, Southampton SO16 6YD, UK; NIHR Southampton Biomedical Research Centre, University Hospital Southampton NHS Foundation Trust and University of Southampton, Southampton SO16 6YD, UK

**Keywords:** polyunsaturated fatty acid, eicosapentaenoic acid, docosahexaenoic acid, metabolism, health, fat-1

## A Perspective on: Comparing Transgenic Production to Supplementation of ω-3 PUFA Reveals Distinct but Overlapping Mechanisms Underlying Protection Against Metabolic and Hepatic Disorders

**Figure 1.**
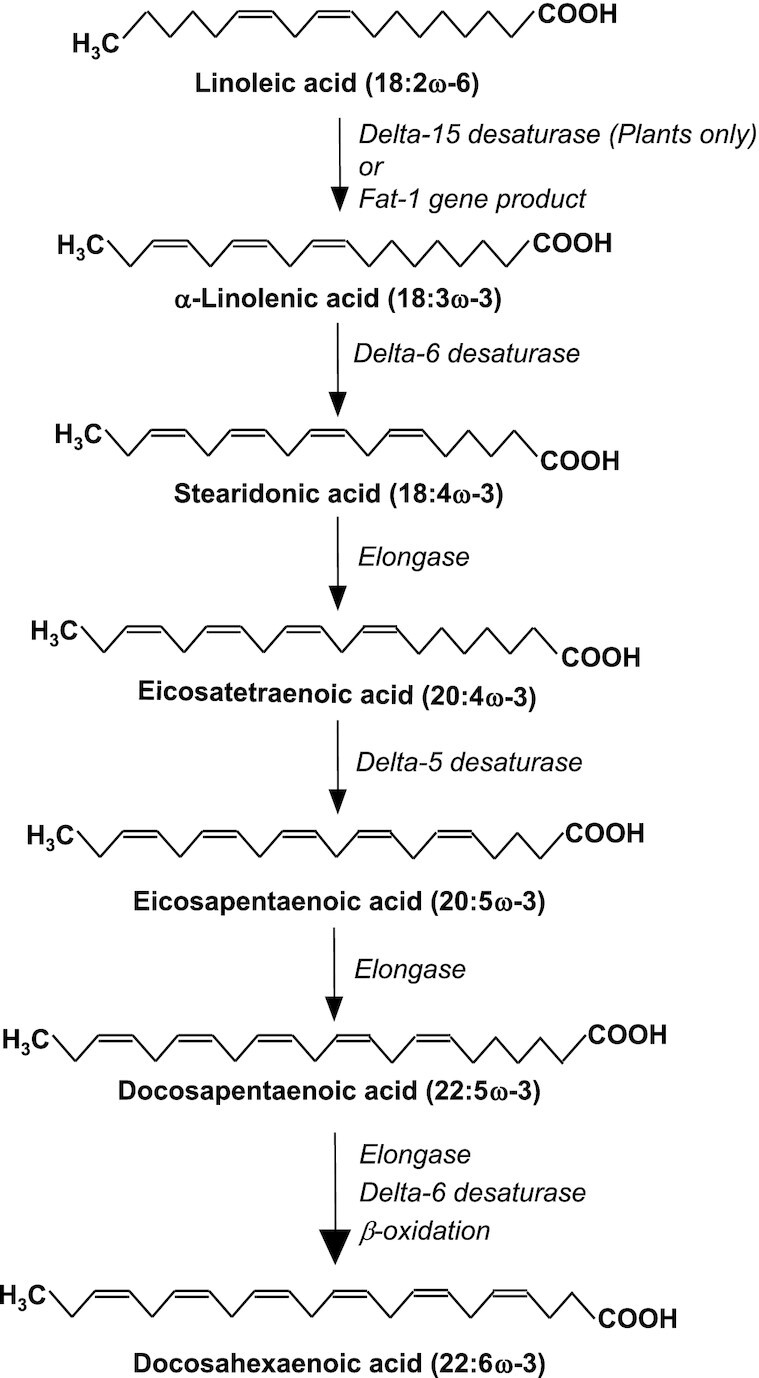
The pathway of conversion of linoleic acid to docosahexaenoic acid.

Omega-3 (ω-3) polyunsaturated fatty acids (PUFAs) are a family of fatty acids distinguished by the presence of the double bond closest to the methyl terminus of the acyl chain being on carbon number 3 counting from the methyl terminal carbon. There are several members of the ω-3 PUFA family. Usually, the most common ω-3 PUFA in the human diet is α-linolenic acid (ALA; 18:3ω-3), an essential fatty acid made in plants from the ω-6 PUFA linoleic acid (LA; 18:2ω-6) by an enzymatic conversion catalyzed by delta-15 desaturase (Figure 1). Animals do not possess the latter enzyme, so they cannot make ALA. Nevertheless, once consumed in the diet, ALA can be converted by animals into long-chain, more unsaturated ω-3 PUFAs, including eicosapentaenoic acid (EPA; 20:5ω-3), docosapentaenoic acid (DPA; 22:5ω-3), and docosahexaenoic acid (DHA: 22:6ω-3) (Figure 1). EPA and DHA are biologically active, influencing cell membrane structure, intracellular signaling pathways, gene expression, and lipid mediator synthesis.^1^ DPA is less well studied but seems to have similar actions to EPA and DHA. Amongst dietary sources, EPA and DHA are found in the highest amounts in fatty fish; they are also present in fish oil-type supplements. EPA and DHA are linked to many health benefits, including reducing the risk of cardiovascular disease and mortality^2^; these effects are due to beneficial modification of a number of risk factors.^3^ There is also evidence that EPA and DHA might reduce the risk of developing nonalcoholic fatty liver disease, through effects on hepatic carbohydrate and fat metabolism and on inflammation.^4^ In general, case-control studies and longitudinal cohort studies provide stronger evidence for the benefits of EPA and DHA on disease outcomes, with findings from randomized controlled trials in patients at risk of, or already with, disease being inconsistent. Circulating and cell and tissue EPA, DPA, and DHA could come directly from the diet or from endogenous biosynthesis starting with ALA as substrate and using the pathway shown in Figure 1. In people with very low or no intake of seafood and not using supplements that contain EPA, DPA, and DHA, it seems likely that much of the body’s EPA, DPA, and DHA are produced through endogenous biosynthesis.^5^ Thus, a major role of ALA is as a precursor to its more bioactive ω-3 PUFA derivatives. Endogenous biosynthesis is likely to be downregulated when there is more EPA, DPA, and DHA in the diet.^6^ However, the relative contributions of diet and endogenous biosynthesis to EPA, DPA, and DHA levels in any compartment or pool within the body are not known. Furthermore, whether the origin of these fatty acids affects their biological action is not well studied. A recent paper published in *Function* starts to address these questions using murine models.^7^

Daniel et al.^7^ use wild-type C57Bl/6 mice and fat-1 mice. The latter are transgenic mice expressing the fat-1 gene from *Caenorhabditis elegans*, which encodes an enzyme with delta-15 desaturase activity, and so able to convert ω-6 into ω-3 PUFAs.^8^ Hence, fat-1 mice fed on diets completely lacking any ω-3 PUFAs have significant amounts of ALA, EPA, DPA, and DHA in their blood, cells, and tissues, unlike wild-type mice fed on such diets. Hence, almost all ω-3 PUFAs in the fat-1 mouse have been endogenously synthesized. Fat-1 mice are well described to have better metabolic characteristics than wild-type mice and reduced incidence and severity of disease in many different murine models of pathology. These findings are consistent with much human research on the longer-chain ω-3 PUFAs like EPA and DHA. In their study, Daniel et al.^7^ fed both types of mouse diets low or high in fat (10% or 55% energy from fat), which provided modest amounts of LA. These experiments allowed the response of wild-type and fat-1 mice to high-fat feeding to be compared. In additional experiments, the authors provided either extra LA or fish oil to the wild-type mice. These experiments allowed (a) the effects of fish oil in wild-type mice to be studied and (b) the effects of ω-3 PUFAs derived from the diet (wild-type mice given fish oil) or produced endogenously (fat-1 mice) to be compared. Multiple outcomes related to metabolic homeostasis and inflammation were measured.

Fat-1 mice fed a high-fat diet had less weight gain; smaller livers; better insulin sensitivity; lower plasma cholesterol; higher hepatic EPA, DPA, and DHA and lower hepatic LA; altered hepatic phospholipid and monoacylglycerol species; selectively lower hepatic cytokines; and modestly altered gut microbiota than seen in the comparator wild-type high-fat group. Including fish oil as part of a high-fat diet in wild-type mice resulted in less weight gain; smaller livers; lower plasma leptin, cholesterol, and triglycerides; lower hepatic triglycerides; higher hepatic EPA, DPA, DHA, 17-hydroxy-DHA, 18-hydroxy-EPA, and protectin DX; lower hepatic palmitic acid, LA, arachidonic acid, leukotriene B_4_, and ceramides; altered hepatic phospholipid and monoacylglycerol species and bile acids; decreased ω-6 PUFA containing hepatic endocannabinoids and increased EPA and DHA containing hepatic endocannabinoids; selectively lower hepatic cytokines; altered gut microbiota; higher caecal weight and propionate concentration; and lower fecal lipopolysaccharide and flagellin than seen in the comparator wild-type high-fat group. Thus, the study confirms (a) a better metabolic state in fat-1 mice fed a high-fat diet than in wild-type mice fed that diet and (b) that dietary fish oil (a source of EPA and DHA) improves the metabolic state of wild-type mice fed a high-fat diet. It is important to note that the detail of the analysis of hepatic and skeletal muscle lipid species described by Daniel et al. is impressive and includes novel observations.

There is commonality in many of the effects observed in fat-1 mice fed a high-fat diet and wild-type mice fed a high-fat diet enriched in fish oil, although the extent of these effects is often seen to be different. For example, feeding fish oil to wild-type mice had a greater effect on hepatic and skeletal muscle endocannabinoids and on hepatic bile acids than seen in fat-1 mice fed a high-fat diet. These quantitative differences most likely relate to the differences observed in hepatic ω-3 PUFA levels achieved in the two models. Fat-1 mice fed the high-fat diet achieved mean hepatic EPA, DPA, and DHA concentrations of ∼12, ∼10, and ∼60 μm, respectively. In contrast, wild-type mice fed fish oil achieved concentrations of ∼80, ∼40, and ∼80 μm for the three fatty acids. These higher concentrations would be expected to exert greater effects on hepatic metabolism. There was also a greater effect of fish oil in wild-type mice on gut microbiota than seen in fat-1 mice, and fat-1 mice did not show increased caecal weight and increased caecal short-chain fatty acids. Again, these differences might relate to the different systemic levels of ω-3 PUFAs achieved, as just described, but they could also relate to the way that the increased ω-3 PUFA status is achieved in the two models. The high-fat-fed wild-type mice received oral fish oil; some of the component fatty acids might escape small intestinal absorption and make their way to the caecum and large intestine where they could have a prebiotic-type action.^9^ In contrast, in fat-1 mice, ω-3 PUFAs are produced endogenously from LA in the liver; thus, the lumen of the intestinal tract in fat-1 mice is not directly exposed to ω-3 PUFAs, meaning a prebiotic effect is unlikely. Fat-1 mice are described to have a different gut microbiota from wild-type mice,^10^ and this might relate to altered fatty acid composition of the gut epithelium and/or to effects of systemic ω-3 PUFAs on the gut-associated immune and inflammatory systems. If this mechanism is important in fat-1 mice, it might be that the lower levels of ω-3 PUFAs achieved in the fat-1 mice studied by Daniel et al.^7^ are too low to have a strong influence on the intestinal tract ecosystem.

While the commonality of effects seen in fat-1 mice fed a high-fat diet and wild-type mice fed a high-fat diet and receiving fish oil is interesting and encouraging about the metabolic actions of ω-3 PUFAs, the different effects seen are intriguing. The differences seen in caecal size and short-chain fatty acids have already been discussed. Additionally, feeding fish oil to wild-type mice as part of a high-fat diet abolished the hepatic steatosis seen in the high-fat control group. In contrast, hepatic steatosis was not different between wild-type and fat-1 mice fed a high-fat diet. There are several possible explanations for this marked difference in impact of dietary and endogenously produced ω-3 PUFAs. One is that this might relate to the differences in gut microbiota that occur with the different experimental approaches. Second is that the difference might again relate to the lower hepatic (and presumably systemic) ω-3 PUFAs achieved in the fat-1 mice compared with the wild-type mice fed fish oil. The hepatic concentrations of these fatty acids in the fat-1 mice might just be too low to affect the molecular and metabolic machinery that results in hepatic triglyceride synthesis and accumulation. On the other hand, the observations could indicate that endogenously produced and dietary ω-3 PUFAs can actually have different effects. This might relate to differences in the way in which fatty acids of different origins are handled. Dietary ω-3 PUFAs enter the body in chylomicrons and are cleared, in part, by adipose tissue, where they are stored, but where they also have biological effects. In this regard, Daniel et al.^7^ show that visceral adipose tissue weight and the plasma concentration of the adipose tissue-derived hormone leptin are lower in wild-type mice fed fish oil than in the comparator group, suggesting that dietary ω-3 PUFAs have direct effects on adipose tissue. ω-3 PUFAs are subsequently delivered to the liver in remnant particles and in a nonesterified form. In this way, they could act as substrates for hepatic complex lipid synthesis (eg, phospholipids, endocannabinoids, which are dramatically altered in high-fat-fed wild-type mice receiving fish oil) and as regulators of hepatic lipid metabolism, so influencing the profile of hepatic bile acids and the pathway of hepatic triglyceride synthesis, the result of the latter effects being reduced hepatic storage of triglycerides. In fat-1 mice, ω-3 PUFAs are produced in the liver, and obviously could have direct hepatic effects and also be exported as components of very low density lipoproteins. The latter are cleared by adipose tissue in an analogous way to chylomicrons, yet fat-1 mice do not show lower visceral adipose tissue or plasma leptin compared to wild-type mice. Thus, in some respects, endogenously produced ω-3 PUFAs behave differently from dietary (ie, exogenously sourced) ω-3 PUFAs, which may relate to subtleties in the differences in metabolic handling and channeling of ω-3 PUFAs of different origins, with this difference likely being exerted in the liver, but perhaps also in adipose tissue.

The study of Daniel et al.^7^ is important because it confirms, using a sophisticated experimental design, the metabolic benefits of ω-3 PUFAs but produces findings that also suggest that endogenously produced and dietary ω-3 PUFAs can have different metabolic effects. The mechanism for this needs further exploration. One important limitation of this work, however, is that different hepatic ω-3 PUFA concentrations were achieved in the two different experimental scenarios (fat-1 mice fed a high-fat diet and wild-type mice fed a high-fat diet with fish oil), meaning that the differences seen might simply relate to an achieved ω-3 PUFA concentration below the concentration needed to exert some biological actions. In this regard, it is important to note that Daniel et al.^7^ use hemizygous fat-1 mice (*fat-1*^+/−)^. It is likely that fat-1 homozygotes and possibly also heterozygotes achieve higher levels of ω-3 PUFAs than hemizygotes. The implications of this research for human metabolism and human nutrition are clear—ω-3 PUFAs of endogenous and exogenous origin may be handled differently and, consequently, have different biological actions or potencies—and may explain some of the differences seen between observational and intervention trials of ω-3 PUFAs.

## Data Availability

This article does not include data.
