# Correction to: Do Endogenously Produced and Dietary ω-3 Fatty Acids Act Differently?

**DOI:** 10.1093/function/zqad052

**Published:** 2023-09-26

This is a correction to: Philip C Calder, Do Endogenously Produced and Dietary ω-3 Fatty Acids Act Differently?, *Function*, Volume 4, Issue 3, 2023, zqad009, https://doi.org/10.1093/function/zqad009

In the originally published version of this manuscript, in the introduction of the article, the author describes the types of essential fatty acids, specifically mentioning the ω-6 PUFA linoleic acid (LA; 18:2ω-3). The sentence is "Usually, the most common ω-3 PUFA in the human diet is α-linolenic acid (ALA; 18:3ω-3), an essential fatty acid made in plants from the ω-6 PUFA linoleic acid (LA; 18:2ω-3) by an enzymatic conversion catalyzed by delta-15 desaturase (Figure 1)."

However, (LA; 18:2ω-3) should be corrected to (LA; 18:2ω-6) because linoleic acid belongs to ω-6 PUFA.

This error has been corrected online.

